# The genome sequence of the Rusty-dot Pearl moth, *Udea ferrugalis *(Hübner, 1796)

**DOI:** 10.12688/wellcomeopenres.21571.1

**Published:** 2024-05-15

**Authors:** David C. Lees, Natural History Museum Genome Acquisition Lab, Darwin Tree of Life Barcoding collective, Wellcome Sanger Institute Tree of Life Management, Samples and Laboratory team, Wellcome Sanger Institute Scientific Operations: Sequencing Operations, Wellcome Sanger Institute Tree of Life Core Informatics team, Tree of Life Core Informatics collective, Darwin Tree of Life Consortium

**Affiliations:** 1 Natural History Museum, London, England, UK

**Keywords:** Udea ferrugalis, Rusty-dot Pearl moth, genome sequence, chromosomal, Lepidoptera

## Abstract

We present a genome assembly from an individual male *Udea ferrugalis* (the Rusty-dot Pearl moth; Arthropoda; Insecta; Lepidoptera; Crambidae). The genome sequence is 495.6 megabases in span. Most of the assembly is scaffolded into 31 chromosomal pseudomolecules, including the Z sex chromosome. The mitochondrial genome has also been assembled and is 15.39 kilobases in length. Gene annotation of this assembly on Ensembl identified 18,035 protein coding genes.

## Species taxonomy

Opisthokonta; Metazoa; Eumetazoa; Bilateria; Protostomia; Ecdysozoa; Panarthropoda; Arthropoda; Mandibulata; Pancrustacea; Hexapoda; Insecta; Dicondylia; Pterygota; Neoptera; Endopterygota; Amphiesmenoptera; Lepidoptera; Glossata; Neolepidoptera; Heteroneura; Ditrysia; Obtectomera; Pyraloidea; Crambidae; Spilomelinae; *Udea*; *Udea ferrugalis* (Hübner, 1796) (NCBI:txid1002954).

## Background

*Udea ferrugalis* (Hübner, 1796) also known as the Rusty-dot Pearl ( Sterling *et al.*, 2023), is a crambid moth with a wingspan/forewing length of 18–24 mm/9–11 mm ( Parsons & Clancy, 2023; Sterling *et al.*, 2023). Distinguishing it from almost all other *Udea* Guenée in Duponchel, 1845, its forewing is a rusty brown colour with faintly marked dark grey orbicular and reniform stigmata, and often a few greyish marks or dots displaced proximally, a finely jagged postdiscal dark grey line convexly curved around the reniform, and a dark grey fringe; the hindwing is pale greyish-translucent with a discal spot and darker scales towards the termen. The legs are white scaled. The Rusty-dot Pearl rests in a triangular shape ( Parsons & Clancy, 2023). The forewings are quite pointed at the apex, more so than the similar *U. delineatalis* (Walker in Meliss, 1875) from St Helena ( Mally *et al.*, 2022).

*U. ferrugalis* is a strongly migratory species, which is also a temporary resident in the United Kingdom, where it is more frequent towards the southern coast. The species is widespread from the Macaronesian islands, throughout Europe from the Mediterranean to northern Scandinavia and through Asia Minor and Asia as far east as Japan, North Africa, India with an isolated record in eastern North America ( GBIF Secretariat, 2024) and throughout the Afrotropics including sub-Saharan Africa and Indian Ocean islands ( De Prins & De Prins, 2023).

Adults can be seen in any month in the UK but more frequently from August to October. They are easily disturbed by day but essentially nocturnal, when they visit a range of flowers ( Parsons & Clancy, 2023).

The yellowish greenish larvae with dark median line and yellowish head are polyphagous on a wide range of herbaceous plants especially species belonging to Solanaceae, Lamiaceae and Asteraceae ( Parsons & Clancy, 2023; see Lepiforum, 2024 for a list). They are continuously brooded in the tropics, but with two generations usually in Europe, where they overwinter as a dark brown shiny pupa in a cocoon within a cut fold of a leaf ( Lepiforum, 2024; Parsons & Clancy, 2023; Sterling *et al.*, 2023).

*U. ferrugalis* forms a single BIN on BOLD (08/04/2024), BOLD:AAC3729, and the mitogenome from the genome assembly (OX465546.1) shares a common haplotype in COI-5P to many DNA barcodes on BOLD. It is 3.47% pairwise divergent from *Udea stellata (Butler, 1883)* (BOLD:ABA1630). *U. delineatalis* and the Asiatic *U. testacea* (Butler, 1879) are placed, though, in the same BIN as *U. ferrugalis* (BOLD:AAC3729) although they are distinguishable morphologically ( Mally *et al.*, 2022). In a morphological study of *Udea* ( Mally & Nuss, 2011), *U. ferrugalis* was recovered as sister to *U. delineatalis* from St Helena among ten species placed in the *U. ferrugalis* species group of island species, as it was in a subsequent combined morphological and molecular study (using COI and Wingless genes) ( Mally *et al.*, 2022).

The genome will be helpful, for example in further molecular taxonomic work on *Udea*. The genome of *Udea ferrugalis* was sequenced as part of the Darwin Tree of Life Project, a collaborative effort to sequence all named eukaryotic species in the Atlantic Archipelago of Britain and Ireland.

## Genome sequence report

**Figure 1.**
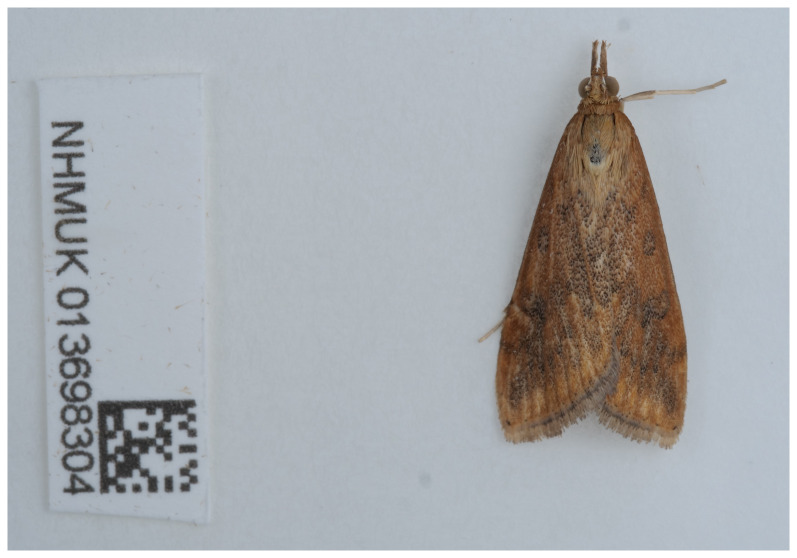
Photograph of the *Udea ferrugalis* (ilUdeFerr1) specimen used for genome sequencing.

The genome was sequenced from one male *Udea ferrugalis* ( Figure 1) collected from Sandwich Bay Bird Observatory, England, UK (51.27, 1.37). A total of 58-fold coverage in Pacific Biosciences single-molecule HiFi long reads was generated. Primary assembly contigs were scaffolded with chromosome conformation Hi-C data. Manual assembly curation corrected 15 missing joins or mis-joins and removed 4 haplotypic duplications, reducing the assembly length by 2.39%.

**Figure 2.**
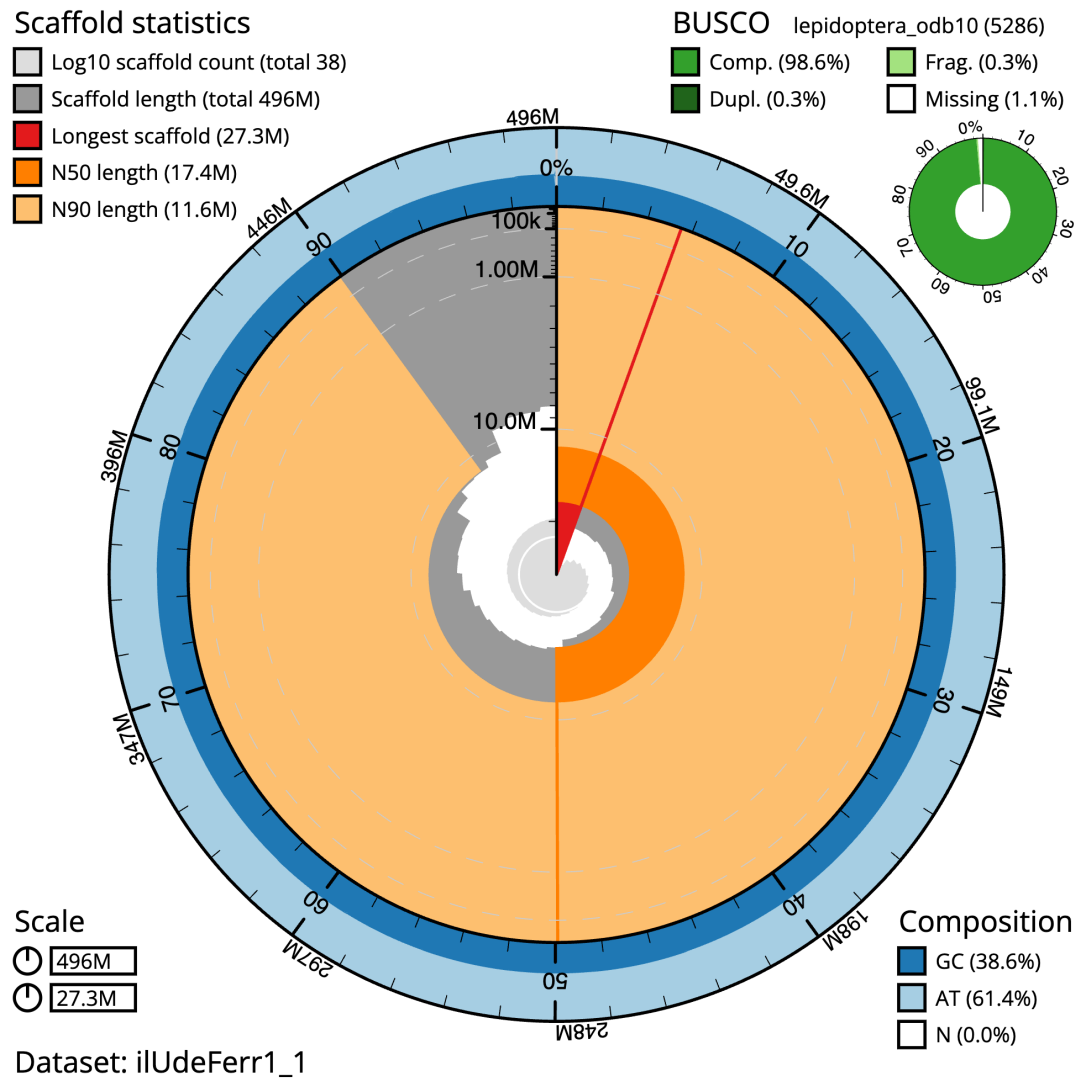
Genome assembly of *Udea ferrugalis*, ilUdeFerr1.1: metrics. The BlobToolKit snail plot shows N50 metrics and BUSCO gene completeness. The main plot is divided into 1,000 size-ordered bins around the circumference with each bin representing 0.1% of the 495,610,244 bp assembly. The distribution of scaffold lengths is shown in dark grey with the plot radius scaled to the longest scaffold present in the assembly (27,305,846 bp, shown in red). Orange and pale-orange arcs show the N50 and N90 scaffold lengths (17,412,294 and 11,621,885 bp), respectively. The pale grey spiral shows the cumulative scaffold count on a log scale with white scale lines showing successive orders of magnitude. The blue and pale-blue area around the outside of the plot shows the distribution of GC, AT and N percentages in the same bins as the inner plot. A summary of complete, fragmented, duplicated and missing BUSCO genes in the lepidoptera_odb10 set is shown in the top right. An interactive version of this figure is available at https://blobtoolkit.genomehubs.org/view/ilUdeFerr1_1/dataset/ilUdeFerr1_1/snail.

**Figure 3.**
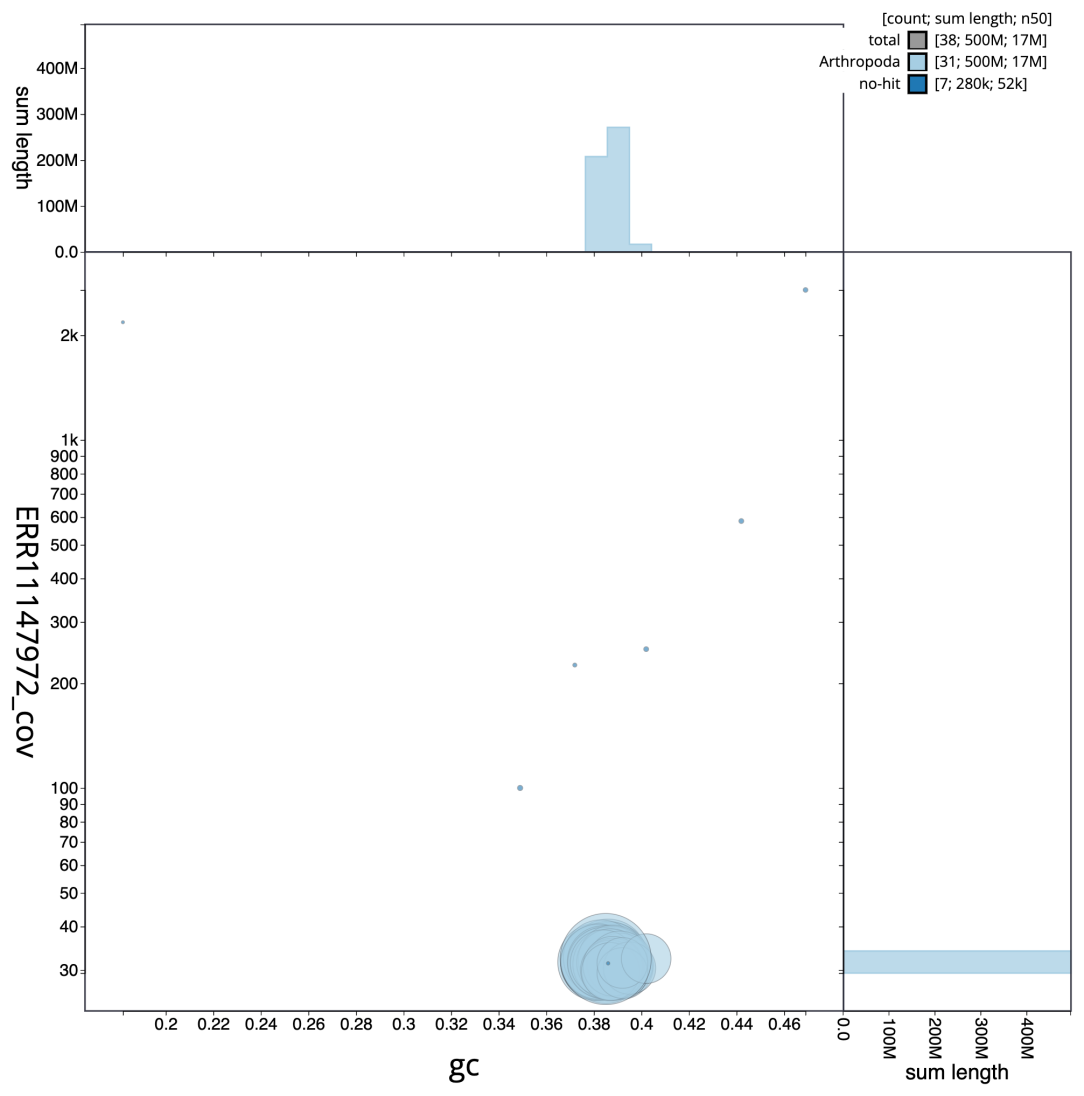
Genome assembly of *Udea ferrugalis*, ilUdeFerr1.1: BlobToolKit GC-coverage plot. Sequences are coloured by phylum. Circles are sized in proportion to sequence length. Histograms show the distribution of sequence length sum along each axis. An interactive version of this figure is available at https://blobtoolkit.genomehubs.org/view/ilUdeFerr1_1/dataset/ilUdeFerr1_1/blob.

**Figure 4.**
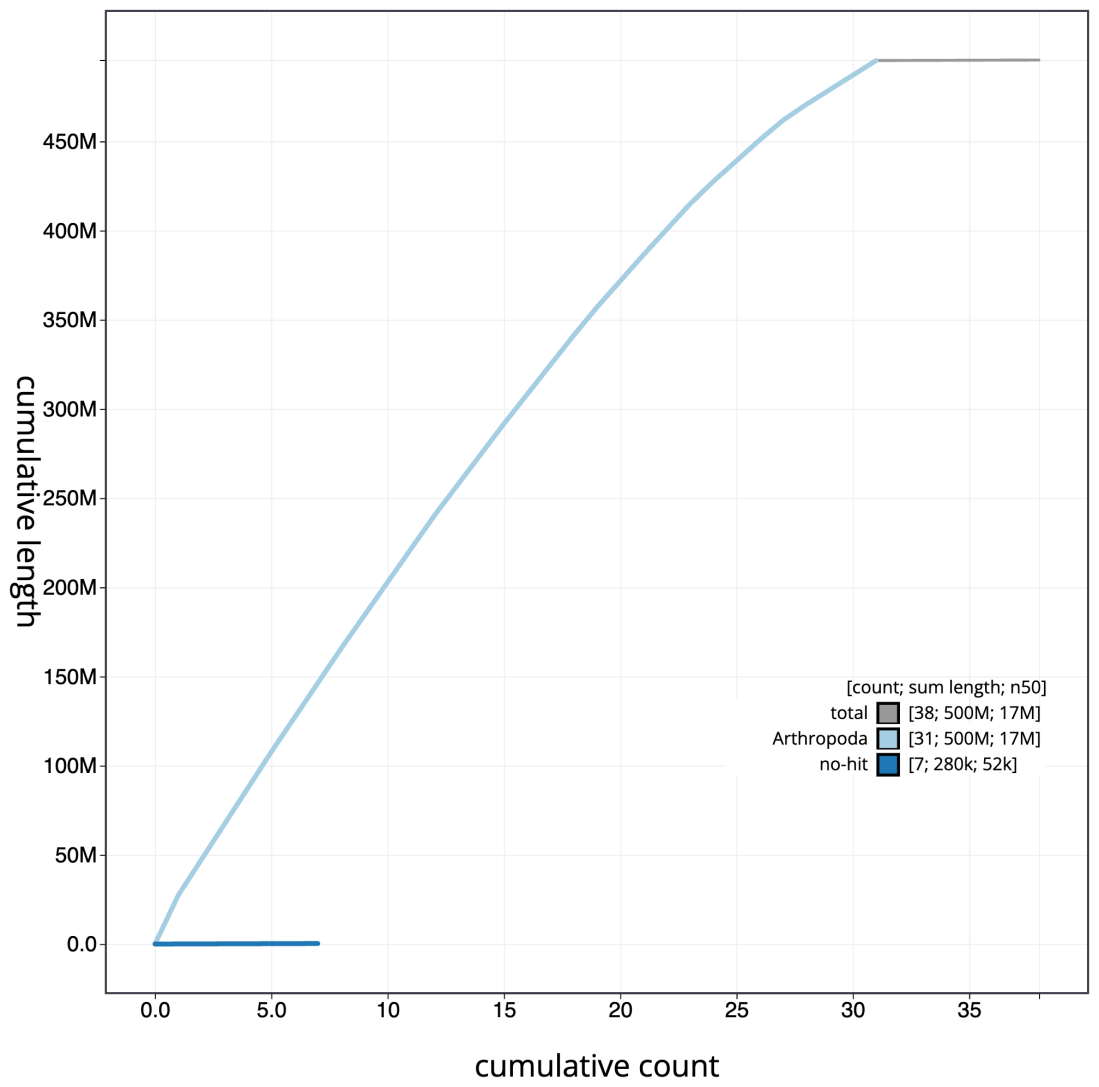
Genome assembly of *Udea ferrugalis*, ilUdeFerr1.1: BlobToolKit cumulative sequence plot. The grey line shows cumulative length for all sequences. Coloured lines show cumulative lengths of sequences assigned to each phylum using the buscogenes taxrule. An interactive version of this figure is available at https://blobtoolkit.genomehubs.org/view/ilUdeFerr1_1/dataset/ilUdeFerr1_1/cumulative.

**Figure 5.**
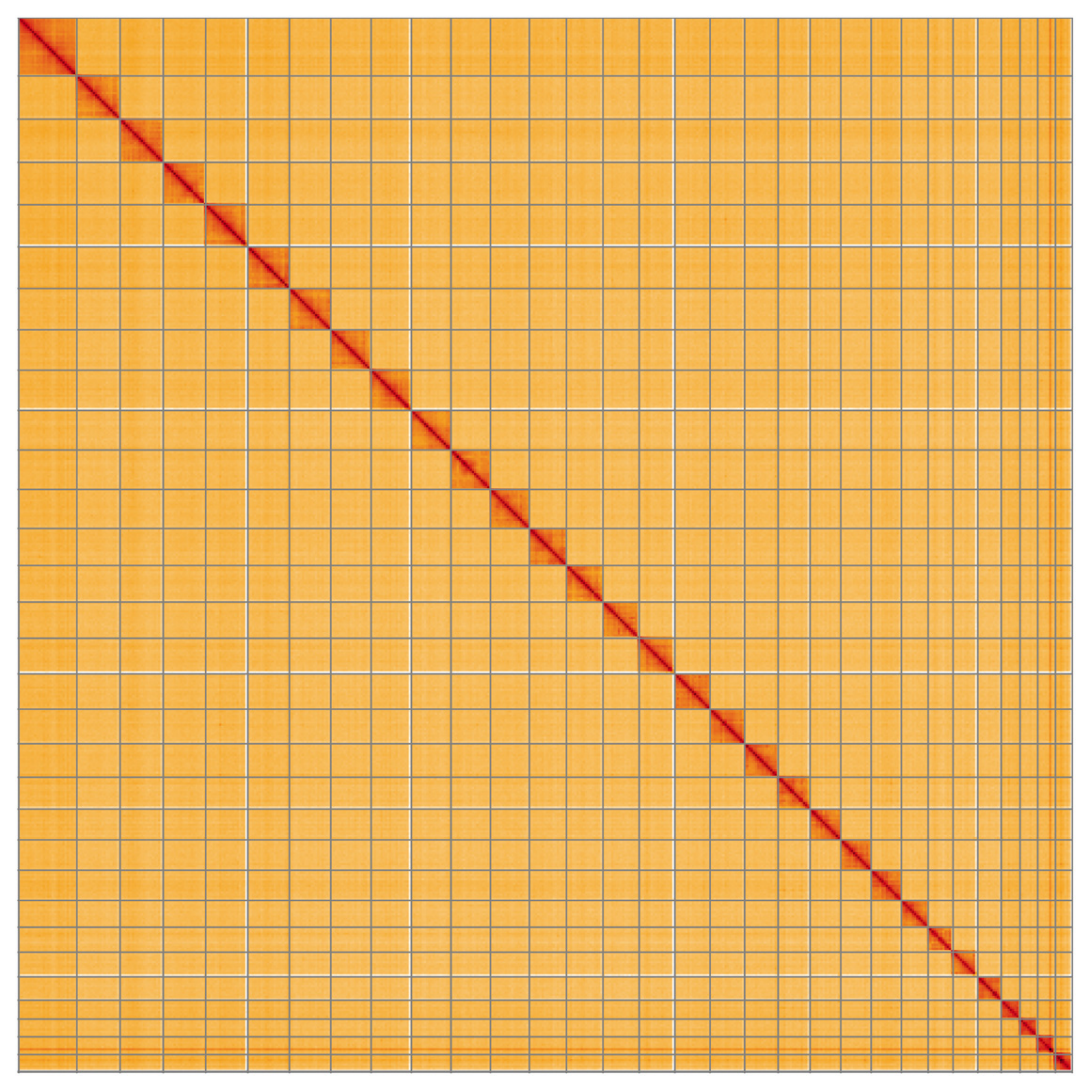
Genome assembly of *Udea ferrugalis*, ilUdeFerr1.1: Hi-C contact map of the ilUdeFerr1.1 assembly, visualised using HiGlass. Chromosomes are shown in order of size from left to right and top to bottom. An interactive version of this figure may be viewed at https://genome-note-higlass.tol.sanger.ac.uk/l/?d=Cct6xLGKSeOg_An1-gaUHw.

The final assembly has a total length of 495.6 Mb in 37 sequence scaffolds with a scaffold N50 of 17.4 Mb ( Table 1). The snail plot in Figure 2 provides a summary of the assembly statistics, while the distribution of assembly scaffolds on GC proportion and coverage is shown in Figure 3. The cumulative assembly plot in Figure 4 shows curves for subsets of scaffolds assigned to different phyla. Most (99.94%) of the assembly sequence was assigned to 31 chromosomal-level scaffolds, representing 30 autosomes and the Z sex chromosome. Chromosome-scale scaffolds confirmed by the Hi-C data are named in order of size ( Figure 5; Table 2). Chromosome Z was assigned by synteny to *Udea olivalis* (GCA_947369235.1) ( Boyes *et al.*, 2023). While not fully phased, the assembly deposited is of one haplotype. Contigs corresponding to the second haplotype have also been deposited. The mitochondrial genome was also assembled and can be found as a contig within the multifasta file of the genome submission.

**Table 1.** Genome data for *Udea ferrugalis*, ilUdeFerr1.1.

Project accession data		
Assembly identifier	ilUdeFerr1.1	
Species	*Udea ferrugalis*	
Specimen	ilUdeFerr1	
NCBI taxonomy ID	1002954	
BioProject	PRJEB60827	
BioSample ID	SAMEA111458572	
Isolate information	ilUdeFerr1, male: head and thorax (DNA and Hi-C sequencing), abdomen (RNA sequencing)	
Assembly metrics *		*Benchmark*
Consensus quality (QV)	70.6	*≥ 50*
*k*-mer completeness	100.0%	*≥ 95%*
BUSCO **	C:98.6%[S:98.3%,D:0.3%],F:0.3%, M:1.1%,n:5,286	*C ≥ 95%*
Percentage of assembly mapped to chromosomes	99.94%	*≥ 95%*
Sex chromosomes	Z	*localised homologous pairs*
Organelles	Mitochondrial genome: 15.39 kb	*complete single alleles*
Raw data accessions		
PacificBiosciences Sequel IIe	ERR11147972	
Hi-C Illumina	ERR11156557	
PolyA RNA-Seq Illumina	ERR12245550	
Genome assembly		
Assembly accession	GCA_950022985.1	
*Accession of alternate haplotype*	GCA_950022945.1	
Span (Mb)	495.6	
Number of contigs	67	
Contig N50 length (Mb)	13.2	
Number of scaffolds	37	
Scaffold N50 length (Mb)	17.4	
Longest scaffold (Mb)	27.31	
Genome annotation		
Number of protein-coding genes	18,035	
Number of gene transcripts	18,307	

* Assembly metric benchmarks are adapted from column VGP-2020 of “Table 1: Proposed standards and metrics for defining genome assembly quality” from Rhie *et al.* (2021). ** BUSCO scores based on the lepidoptera_odb10 BUSCO set using version 5.3.2. C = complete [S = single copy, D = duplicated], F = fragmented, M = missing, n = number of orthologues in comparison. A full set of BUSCO scores is available at https://blobtoolkit.genomehubs.org/view/ilUdeFerr1_1/dataset/ilUdeFerr1_1/busco.

**Table 2.** Chromosomal pseudomolecules in the genome assembly of *Udea ferrugalis*, ilUdeFerr1.

INSDC accession	Chromosome	Length (Mb)	GC%
OX465516.1	1	20.33	38.5
OX465517.1	2	20.27	38.5
OX465518.1	3	19.97	38.5
OX465519.1	4	19.81	38.5
OX465520.1	5	19.57	38.5
OX465521.1	6	19.43	38.0
OX465522.1	7	18.97	38.0
OX465523.1	8	18.92	38.0
OX465524.1	9	18.61	38.5
OX465525.1	10	18.54	38.0
OX465526.1	11	18.32	38.5
OX465527.1	12	17.41	38.5
OX465528.1	13	17.18	38.5
OX465529.1	14	17.04	38.5
OX465530.1	15	16.76	39.0
OX465531.1	16	16.55	38.5
OX465532.1	17	16.26	38.5
OX465533.1	18	15.75	39.0
OX465534.1	19	15.0	38.5
OX465535.1	20	14.43	39.0
OX465536.1	21	14.29	38.5
OX465537.1	22	14.19	39.0
OX465538.1	23	12.6	39.0
OX465539.1	24	11.73	38.5
OX465540.1	25	11.62	39.0
OX465541.1	26	10.97	38.5
OX465542.1	27	8.88	39.5
OX465543.1	28	8.33	39.0
OX465544.1	29	8.32	39.0
OX465545.1	30	7.96	40.0
OX465515.1	Z	27.31	38.5
OX465546.1	MT	0.02	18.5

The estimated Quality Value (QV) of the final assembly is 70.6 with *k*-mer completeness of 100.0%, and the assembly has a BUSCO v5.3.2 completeness of 98.6% (single = 98.3%, duplicated = 0.3%), using the lepidoptera_odb10 reference set ( *n* = 5,286).

Metadata for specimens, BOLD barcode results, spectra estimates, sequencing runs, contaminants and pre-curation assembly statistics are given at https://links.tol.sanger.ac.uk/species/1002954.

## Genome annotation report

The *Udea ferrugalis* genome assembly (GCA_950022985.1) was annotated at the European Bioinformatics Institute (EBI) on Ensembl Rapid Release. The resulting annotation includes 18,307 transcribed mRNAs from 18,035 protein-coding genes ( Table 1; https://rapid.ensembl.org/Udea_ferrugalis_GCA_950022985.1/Info/Index).

## Methods

### Sample acquisition and nucleic acid extraction

A male *Udea ferrugalis* (specimen ID NHMUK013698304, ToLID ilUdeFerr1) was hand-picked from Sandwich Bay Bird Observatory, England, UK (latitude 51.27, longitude 1.37) on 2021-09-24. The specimen was collected and identified by David Lees (Natural History Museum) and preserved by dry freezing at –80 °C.

The workflow for high molecular weight (HMW) DNA extraction at the Wellcome Sanger Institute (WSI) Tree of Life Core Laboratory includes a sequence of core procedures: sample preparation; sample homogenisation, DNA extraction, fragmentation, and clean-up. The sample was prepared for DNA extraction at the WSI Tree of Life Core Laboratory. The ilUdeFerr1 sample was weighed and dissected on dry ice ( Jay *et al.*, 2023) and head and thorax tissue was homogenised using a PowerMasher II tissue disruptor ( Denton *et al.*, 2023a), setting aside tissue for Hi-C sequencing.

HMW DNA was extracted in the WSI Scientific Operations core using the Automated MagAttract v2 protocol ( Oatley *et al.*, 2023). The DNA was sheared into an average fragment size of 12–20 kb in a Megaruptor 3 system with speed setting 31 ( Bates *et al.*, 2023). Sheared DNA was purified by solid-phase reversible immobilisation ( Strickland *et al.*, 2023): in brief, the method employs a 1.8X ratio of AMPure PB beads to sample to eliminate shorter fragments and concentrate the DNA. The concentration of the sheared and purified DNA was assessed using a Nanodrop spectrophotometer and Qubit Fluorometer and Qubit dsDNA High Sensitivity Assay kit. Fragment size distribution was evaluated by running the sample on the FemtoPulse system.

RNA was extracted from abdomen tissue of ilUdeFerr1 in the Tree of Life Laboratory at the WSI using the RNA Extraction: Automated MagMax™ *mir*Vana protocol ( do Amaral *et al.*, 2023). The RNA concentration was assessed using a Nanodrop spectrophotometer and a Qubit Fluorometer using the Qubit RNA Broad-Range Assay kit. Analysis of the integrity of the RNA was done using the Agilent RNA 6000 Pico Kit and Eukaryotic Total RNA assay.

Protocols developed by the WSI Tree of Life laboratory are publicly available on protocols.io ( Denton *et al.*, 2023b).

### Sequencing

Pacific Biosciences HiFi circular consensus DNA sequencing libraries were constructed according to the manufacturers’ instructions. Poly(A) RNA-Seq libraries were constructed using the NEB Ultra II RNA Library Prep kit. DNA and RNA sequencing was performed by the Scientific Operations core at the WSI on Pacific Biosciences Sequel IIe (HiFi) and Illumina NovaSeq 6000 (RNA-Seq) instruments. Hi-C data were also generated from remaining head and thorax tissue of ilUdeFerr1 using the Arima2 kit and sequenced on the Illumina NovaSeq 6000 instrument.

### Genome assembly, curation and evaluation

Assembly was carried out with Hifiasm ( Cheng *et al.*, 2021) and haplotypic duplication was identified and removed with purge_dups ( Guan *et al.*, 2020). The assembly was then scaffolded with Hi-C data ( Rao *et al.*, 2014) using YaHS ( Zhou *et al.*, 2023). The assembly was checked for contamination and corrected as described previously ( Howe *et al.*, 2021). Manual curation was performed using, HiGlass ( Kerpedjiev *et al.*, 2018) and PretextView ( Harry, 2022). The mitochondrial genome was assembled using MitoHiFi ( Uliano-Silva *et al.*, 2023), which runs MitoFinder ( Allio *et al.*, 2020) or MITOS ( Bernt *et al.*, 2013) and uses these annotations to select the final mitochondrial contig and to ensure the general quality of the sequence.

A Hi-C map for the final assembly was produced using bwa-mem2 ( Vasimuddin *et al.*, 2019) in the Cooler file format ( Abdennur & Mirny, 2020). To assess the assembly metrics, the *k*-mer completeness and QV consensus quality values were calculated in Merqury ( Rhie *et al.*, 2020). This work was done using Nextflow ( Di Tommaso *et al.*, 2017) DSL2 pipelines “sanger-tol/readmapping” ( Surana *et al.*, 2023a) and “sanger-tol/genomenote” ( Surana *et al.*, 2023b). The genome was analysed within the BlobToolKit environment ( Challis *et al.*, 2020) and BUSCO scores ( Manni *et al.*, 2021; Simão *et al.*, 2015) were calculated.

Table 3 contains a list of relevant software tool versions and sources.

**Table 3.** Software tools: versions and sources.

Software tool	Version	Source
BlobToolKit	4.2.1	https://github.com/blobtoolkit/blobtoolkit
BUSCO	5.3.2	https://gitlab.com/ezlab/busco
Hifiasm	0.16.1-r375	https://github.com/chhylp123/hifiasm
HiGlass	1.11.6	https://github.com/higlass/higlass
Merqury	MerquryFK	https://github.com/thegenemyers/MERQURY.FK
MitoHiFi	3	https://github.com/marcelauliano/MitoHiFi
PretextView	0.2	https://github.com/wtsi-hpag/PretextView
purge_dups	1.2.5	https://github.com/dfguan/purge_dups
sanger-tol/genomenote	v1.0	https://github.com/sanger-tol/genomenote
sanger-tol/readmapping	1.1.0	https://github.com/sanger-tol/readmapping/tree/1.1.0
YaHS	1.2a.2	https://github.com/c-zhou/yahs

### Genome annotation

The BRAKER2 pipeline ( Brůna *et al.*, 2021) was used in the default protein mode to generate annotation for the *Udea ferrugalis* assembly (GCA_950022985.1) in Ensembl Rapid Release at the EBI.

### Wellcome Sanger Institute – Legal and Governance

The materials that have contributed to this genome note have been supplied by a Darwin Tree of Life Partner. The submission of materials by a Darwin Tree of Life Partner is subject to the **‘Darwin Tree of Life Project Sampling Code of Practice’**, which can be found in full on the Darwin Tree of Life website here. By agreeing with and signing up to the Sampling Code of Practice, the Darwin Tree of Life Partner agrees they will meet the legal and ethical requirements and standards set out within this document in respect of all samples acquired for, and supplied to, the Darwin Tree of Life Project.

Further, the Wellcome Sanger Institute employs a process whereby due diligence is carried out proportionate to the nature of the materials themselves, and the circumstances under which they have been/are to be collected and provided for use. The purpose of this is to address and mitigate any potential legal and/or ethical implications of receipt and use of the materials as part of the research project, and to ensure that in doing so we align with best practice wherever possible. The overarching areas of consideration are:

•   Ethical review of provenance and sourcing of the material

•   Legality of collection, transfer and use (national and international)

Each transfer of samples is further undertaken according to a Research Collaboration Agreement or Material Transfer Agreement entered into by the Darwin Tree of Life Partner, Genome Research Limited (operating as the Wellcome Sanger Institute), and in some circumstances other Darwin Tree of Life collaborators.

## Data Availability

European Nucleotide Archive: *Udea ferrugalis*. Accession number PRJEB60827; https://identifiers.org/ena.embl/PRJEB60827 ( Wellcome Sanger Institute, 2023). The genome sequence is released openly for reuse. The *Udea ferrugalis* genome sequencing initiative is part of the Darwin Tree of Life (DToL) project. All raw sequence data and the assembly have been deposited in INSDC databases. Raw data and assembly accession identifiers are reported in Table 1.

## Author information

Members of the Natural History Museum Genome Acquisition Lab are listed here: https://doi.org/10.5281/zenodo.7139035.

Members of the Darwin Tree of Life Barcoding collective are listed here: https://doi.org/10.5281/zenodo.4893703.

Members of the Wellcome Sanger Institute Tree of Life Management, Samples and Laboratory team are listed here: https://doi.org/10.5281/zenodo.10066175.

Members of Wellcome Sanger Institute Scientific Operations: Sequencing Operations are listed here: https://doi.org/10.5281/zenodo.10043364.

Members of the Wellcome Sanger Institute Tree of Life Core Informatics team are listed here: https://doi.org/10.5281/zenodo.10066637.

Members of the Tree of Life Core Informatics collective are listed here: https://doi.org/10.5281/zenodo.5013541.

Members of the Darwin Tree of Life Consortium are listed here: https://doi.org/10.5281/zenodo.4783558.
