# Supplementary material for: The genome sequence of the Rusty-dot Pearl moth, Udea ferrugalis (Hübner, 1796)
Source: Wellcome Open Res. 2024 May 15;9:259. [Version 1] doi: 10.12688/wellcomeopenres.21571.1 (PMC11452769; doi:10.12688/wellcomeopenres.21571.1)
Supplement: Peer review history

# Reviewer report: Reviewer response for version 1

**DOI:** 10.21956/wellcomeopenres.23843.r99630

**Published:** 2024-10-04

**Authors:** Saurabh Chaudhary

**Affiliations:** 1 The University of Sheffield, Sheffield, England, UK

The genome and mitochondrial genome assemblies of the Rusty-dot Pearl moth, *Udea ferrules*, presented in the current article, show a good-quality genome sequence up to the Darwin Tree of Life Consortium standard. The sequencing strategy and computational tools used in the study are standardised and applied very efficiently.

 Authors utilised the latest Pacific Biosciences single-molecule HiFi long reads to sequence the Pearl moth genome. They also employed appropriate bioinformatics tools such as Hifiasm, HiGlass, PretextView, MitoHiFi, MitoFinder, bwa-mem2, and others. The final genome analysis was performed by the BlobToolKit and BUSCO scores.

 All the figures and tables are representative and explanatory, making the article well-readable and understandable. I am delighted with the genome and mitochondrial genome sequence assembled for *Udea ferrules* in the current research article and highly recommend it for publication.

Are sufficient details of methods and materials provided to allow replication by others?

Yes

Is the rationale for creating the dataset(s) clearly described?

Yes

Are the datasets clearly presented in a useable and accessible format?

Yes

Are the protocols appropriate and is the work technically sound?

Yes

Reviewer Expertise:

Transcriptomics and Genomics of model and non-model species.

I confirm that I have read this submission and believe that I have an appropriate level of expertise to confirm that it is of an acceptable scientific standard.

# Reviewer report: Reviewer response for version 1

**DOI:** 10.21956/wellcomeopenres.23843.r93202

**Published:** 2024-08-29

**Authors:** Melody Clark

**Affiliations:** 1 Natural Environment Research Council, Cambridge, UK

This is a standard format genome note for the Rusty-dot pearl moth, which is a transient visitor to the UK. The morphology and taxonomy of this species are well described. This genome sequence (and mitogenome) will be very useful for molecular taxonomy of *Udea *species in the future. The DNA extraction, sequencing, assembly and analysis protocols are standard for the Darwin Tree of Life Project, are of very high quality and highly reproducible. This genome assembly methodology is reflected in the high quality of this genome.

Are sufficient details of methods and materials provided to allow replication by others?

Yes

Is the rationale for creating the dataset(s) clearly described?

Yes

Are the datasets clearly presented in a useable and accessible format?

Yes

Are the protocols appropriate and is the work technically sound?

Yes

Reviewer Expertise:

Transcriptomics and genomics of non-model species.

I confirm that I have read this submission and believe that I have an appropriate level of expertise to confirm that it is of an acceptable scientific standard.
